# Book Review

**Published:** 1870-08

**Authors:** 


### Books Review

*Third Annual Report of the Metropolitan Board of Health.*

This invaluable report should be in the hands of every physician, indeed of every citizen. We will not now occupy space in a general description of the numerous subjects discussed, but will quote a single paragraph of immense interest to the inhabitants of cities, and hope hereafter to speak of the report more fully. We have also on our table the Report of 1869. The Metropolitan Board of Health are conferring upon humanity an unspeakable blessiug in their reports upon Sanitary Science.

### THE APPLICATION OF DISINFECTANTS.

The summer of 1869 was remarkable for its continued high heat and great humidity, conditions favorable to the rapid changes of organic matter, and very unfavorable to the health of cities not thoroughly and daily cleansed of all filth. After nearly a month of excessively damp and hot weather, the death rate of New Yoık began to increase in the early part of July at such a rate as to awaken public anxiety. In the week ending July 11th there were 614 deaths; the mean temperature had been eighty degrees for more than two weeks, and the atmosphere was excessively damp, and the earth and all surface filth were completely saturated with moisture. In the following week ending July 18th, the heat increased to eighty-eight degrees, and the weekly mortality mounted up to 1,142, or nearly to that of the fatal week which ushered in the cholera of 1866.

While the death rate was somewhat increased by the direct effects of heat, the reports of the Registrar of Vital Statistics showed that the excess of mortality was largely, if not principally, due to diarrhœal diseases, and further investigation proved that the highest sickness and death rate was in those localities where there was the largest accumulations of street, alley and house filth. In many sections of the city low forms of vegetable growth covered the stagnant and putrescent pools of water, and lined the gutters, curbstones and alleys. At this period also an increasing number of sudden deaths with choleraic symptoms were reported by the Registrar, and the evidences of the immediate danger of an outbreak of cholera were so apparent to the army medical officers that all recruiting was suspended, and the movement of troops in and through New York prohibited. To meet the exigencies of the public health the following measures were adopted by the Board.

1. The more thorough and repeated cleansing and disinfection of all sources of domestic filth by citizens was recommended. To accomplish this object the Board ordered the publication of a tract containing simple rules for the use of disinfectants prepared by the Registrar, Dr. Harris. This tract was widely circulated and very many of our citizens faithfully followed the advice given.

2. The more thorough and efficient employment of disinfectants by scavengers in removing the contents of privies, and subsequently the entire cessation of the work of scavenging except in urgent cases.

3. The application of most powerful disinfectants to the streets and gutters. Up to this period the only disinfectant employed for street gutters and surface filth had been chloride of lime. This material had already been freely used in the most filthy streets, but owing to its feeble antiseptic properties and the difficulty of so applying it as to spread it effectually over large surfaces, and to make it penetrate masses of filth, the Board decided to resort to carbolic acid and copperas, which were recommended by the chemist, Prof. Chandler, as effectual disinfectants for such general use known to sanitary chemistry.

The work of street disinfection was begun on the 24th of July, and prosecuted at first with a large number of carts both night and day. The wards selected were the eleventh and twentieth, but afterward the work was extended to other wards where diarrhoeal diseases were most prevalent. It was continued for about two weeks with a diminished force, and was suspended by the occurrence of heavy rains.

The effect of the disinfectants upon the organic matter of the streets where they were employed was proven by Prof. Chandler to be the complete arrest of all putrescent changes, and this effect continues for upward of a week, or until another layer of filth was deposited. The people of the districts where the sprinkling was done spoke approvingly of the measure, and expressed their pleasure at the change of the odors of street filth to that of carbolic acid.

The most important result of this work, however, is seen in the mortality records of diarrhoeal diseases as exhibited by the records of the Registrar:

| | |
|---|---|
| The total number of diarrhœal diseases in the week ending July 4th, was, | 51 |
| The total number of diarrhœal diseases in the week ending July 11th, was, | 176 |
| The total number of diarrhœal diseases in the week ending July 18th, was, | 416 |
| The total number of diarrhœal diseases in the week ending July 25th, was, | 366 |
| The total number of diarrhœal diseases in the week ending Aug. 1st, was, | 345 |
| The total number of diarrhœal diseases in the week ending Aug. 8th, was, | 325 |
| The total number of diarrhœal diseases in the week ending Aug. 15th, was, | 255 |

From this table it appears that there was a steady decline of deaths from that class of diseases produced by sceptic organic matter, from the commencement of the larger employment of disinfectants.

In reviewing this work and its results, the Board has reason to believe, that an important step has been taken in this country toward controlling a source of unhealthiness, which sanitary authorities, as in this city, frequently have no power to remove. Although, from the limited territory to which disinfectants were applied, the necessarily imperfect methods of distribution in this first effort, and the brief period during which the work was continued, the results as regards the effects upon mortality are not as demonstrative as could be desired, yet they were sufficiently marked to prove the value and necessity of the work."

*Facts and Remarks concerning Idiocy.* By EDWARD SEGUIN, M. D.

This author treats his subject in a masterly manner; the address is full of truth, philosophy and sense: the best compliment we can give it. Read what he says in his first paragraph on the causes of mental idiocy.

Then, what is the matter? The gist of the matter seems to be: " Betier," said Enfantin and J. S. Mill, " if women could tell it themselves;" that with more subjects of gratification of mind and body to-day than in the past centuries, women are uneasy, unhappy, because they do not feel themselves adequate to their task. Their education—a jumble of that which has made all the male *inutilities* we have known—has not taught them an iota of womanhood. Their hygiene and habits have disqualified them for motherly functions; city and house narrowness do not offer more room for a new-comer than their slender pelves; their tastes rūn toward niceties incompatible with married life; fecundation is the result of maladroitness; its product, unwelcome, ill-fed, ill-treated before as after birth, conceived in apprehension, remains a nervous ruin, or disappears in a storm of some sort. At this spectacle we can sorrow, but not wonder. Can we expect woman to know what she has not learned, or to resent feelings whose warmth never descended into herself? How, besides can she conceive and nurture, with a living enthusiasm, a child she has no strength to carry, no room to grow, no substance to feed, no idea how it is to be handled, cared for, etc.? The heaviest task when it is not the dearest, she shifts it off, coming out from the struggle with a sad countenance and emaciations foreboding early degeneracy of some vital organs. To be frank, we physicians, teachers and parents are more culpable than herself.

---

*History of nine cases of Ovariotomy.* By Prof. T. GAILLARD THOMAS, M. D.

The first case was a large single cyst, of eighteen months standing: recovered. The second case was believed to be ovarian tumor, by all physicians who examined it in connection with Dr. Thomas. The operation was made in Bellevue hospital, in presence of Drs. Peaslee, Budd, Loomis, and the House-staff. It proved to be a fibrocystic tumor, weighing seventeen pounds. Patient died in forty hours from shock. On two other occasions he has been similarly deceived; one in connection with Dr. Peaslee, the other, Dr. O. Reilly. The third case proved to be Alveolar Cancer of left ovary. Patient died on the eighth day, from Septicæmia. Case fourth was multilocular ovarian cyst; pedicle ligated with silk, and returned into abdominal cavity. Patient recovered. The fifth and sixth cases were similar, only the pedicle was treated with clamp. The seventh case was a multilocular tumor; four fifths of the sac removed: pedicle and ligatures left in vagina: death from peritonitis. The eighth case proved to be cancer of both ovaries; pedicles treated by actual cautery: death from peritonitis . The last case was a multilocular cyst, containing many large cysts; pedicle treated by clamp: recovery.

These reports are interesting and instructive in the highest degree, and physicians interested in the subject of ovaritomy will be under many obligations for the details of the conditions, modes of treatment, and results in these cases.

---

*Specialism in its relations to practical Medicine.* By G. S. HUBBARD, M. D., New Haven, Conn.

In a most truthful and well written paper on this subject, read before the Connecticut Medical Society, Dr. Hubbard says:

"Within the last twenty or thirty years, practical medicine in this country has made greater advance in all its departments, than in the two generations previous; in consequence, as I believe, of the superior quality and wider range of the medical education afforded, and the greater demands made upon medical men by a higher civilization—but mainly because of the assiduous cultivation of distinct sub-divisions of medical science, by men who, in greater numbers, have devoted themselves exclusively to the study of them.

The mature results of their labors have been freely given to the profession in systematic treatises, monographs, and papers of less pretension, and as freely appropriated and assimilated by us, until they have become a very large and important portion of the common stock of professional knowledge.—Nearly all the important improvements in operative surgery, the accepted methods of treating diseases of the eye and ear—the skin, the kidneys—diseases of the nervous system, and of the thoracic organs—diseases of the male and female generative organs—the best means of exploring cavities, and indeed nearly our entire knowledge of the structure and physiology of the organs themselves, we owe to the labors, often unrewarded, of a class of men, who, having devoted themselves to the study of a single department, or a subdivision of it, are properly called "specialists."

---

*Medico-Legal Study of the case of Daniel McFarland.* By WILLIAM A. HAMMOND, M. D.

We had supposed that the plea of insanity by McFarland was a mere "dodge," but the following paragraph from this pamphlet leads us to think it might have been founded in some truth. Dr. Hammond, in connection with his study of McFarland's case has furnished a valuable paper upon Insanity. But to the evidence of insanity.

"It is in evidence that the accused, who was a married man, was devotedly and passionately attached to his family; that he had intercepted a letter from the deceased to his wife, which was calculated from its sentiments to arouse the most powerful emotions in the human mind; that his wife had left him, taking with her both the children; that he had instituted legal proceeding to obtain the possession of his offspring; that he was opposed by his wife and deceased, the latter supplying the funds for the resistance of the father's efforts; that these troubles partially unsettled his reason, so that several persons who knew him and were thrown into contact with him remarked that he was incoherent, rambling, excited, and the thought of his domestic difficulties was almost continually present, as shown by his conversation and actions; that he was unable to sleep; that he wandered through the streets at night in all kinds of weather, talking of his troubles to policemen and others; that he could not by reason of his mental condition perform properly the duties of the office he held under the Government of the United States; that various powerful medicines, such as morphia, Indian hemp, hyoscyamus, and bromide of potassium, had been prescribed for him in large doses by his medical attendants; that for several days previous to the homicide he had taken large quantities of morphia; that during this period, and even before, his pulse was never below 104 per minute, and was frequently much more rapid; that his face was flushed, that there was involuntary twitching of the facial muscles; that his eyes were suffused and his pupils contracted; that he had flashes of light and dark specks before his eyes; that he suffered from vertigo; that his head was painful and hot; that he had frequent outbursts of excitement; that he had hallucinations and delusions; that he had doubts as to his identity; that he had threatened to commit suicide; that his memory was impaired; that while in this condition he heard that a divorce had been granted to his wife in the State of Indiana, on *ex parte* statements; that the symptoms of mental disorder then became greatly aggravated; and that on the afternoon of the homicide he was met in the street by a friend who remarked his wild expression, and who was convinced that he was not in his right mind.

"It is also in evidence that a first-cousin of the accused died insane, and that the resemblance of the latter to him in features and manner is very great."

---

*Report of the Committee on the relations of Alcohol to medicine.* By JOHN BELL, M. D., Chairman.

This report is probably the best, most truthful and trustworthy statement of the total uselessness of alcohol as food or medicine ever published. Physicians have endorsed it as useful in low forms of disease in consumption, etc., etc., but they have thus conferred upon mankind a greater curse than they are ever likely to prove a blessing. Where it has relieved or benefited one patient, it has proved more injurious and dangerous to thousands than the malady for which it was prescribed. This report should be placed in the hands of every physician, and indeed of every body, since it shows how wholly useless for good alcohol may be considered to be. Spirituous and fermented liquors exceed in their ill effects upon mankind all other maladies combined, and cause more misery, disease and death. To annihilate it from the world would be a blessing, which we hope this committee and Dr. John Bell, in particular, may live to see bestowed.

*Medical opinion of* CHARLES A. LEE, M. D. Case of CARLTON YATES.

This pamphlet, of about thirty pages, contains a complete *resume* of the law and evidence on the case of Carlton Yates, together with the conclusions of the author, Professor Charles A. Lee, who says that his opinion has been deliberately and conscientiously formed, from a knowledge of the entire testimony offered in this case, all of which has been heard or carefully read; also from intimate acquaintance of over twenty years. He says that Carlton Yates labored under confirmed insanity for at least the last year of his life, and probably for a much longer period; that the form of mental disease was that which usually goes under the name of *monomania*. The article comprises a very careful, comprehensive, and well arranged review of the case, and will be regarded as a valuable contribution to Medico-Legal insantty.

